# Absence of DNA viruses in ameloblastomas

**DOI:** 10.1111/eos.70092

**Published:** 2026-04-10

**Authors:** Maria K. Jauhiainen, Jetta Kelppe, Maria Söderlund‐Venermo, Antti A. Mäkitie, Saku T. Sinkkonen, Jaana Hagström

**Affiliations:** ^1^ Department of Otorhinolaryngology—Head and Neck Surgery, Head and Neck Center Helsinki University Hospital and University of Helsinki Helsinki Finland; ^2^ Department of Virology University of Helsinki and Helsinki University Hospital Helsinki Finland; ^3^ Research Program in Systems Oncology Faculty of Medicine University of Helsinki Helsinki Finland; ^4^ Department of Pathology Helsinki University Hospital Helsinki Finland; ^5^ Division of Ear, Nose, and Throat Diseases, Department of Clinical Sciences Intervention and Technology Karolinska Institute and Karolinska University Hospital Stockholm Sweden; ^6^ Department of Oral Pathology and Radiology University of Turku Turku Finland

**Keywords:** ameloblastoma, DNA viruses, Epstein‐Barr virus, herpesvirus, odontogenic tumor, parvovirus, polyomavirus, viruses

## Abstract

Ameloblastomas (ABs) are rare odontogenic benign tumors. Although studies of the molecular pathogenesis have revealed the presence of mutations and altered gene expression, the etiology is unresolved. Viruses cause 10%–15 % of cancers, but their potential role within benign tumors is less studied. This study focuses on the potential presence of herpes‐, parvo‐, and polyomaviruses in ABs. Thirty DNA viruses comprising herpes‐, parvo‐, and polyomaviruses were analyzed by qPCR and Luminex‐based methods in 11 formalin‐fixed paraffin‐embedded AB samples. In repeated analyses, the ABs harbored none of the studied large set of viruses. Based on our findings a relevant role of the studied viruses seems unlikely in the pathogenesis of ABs.

Ameloblastoma (AB) is a benign but locally aggressive epithelial odontogenic tumor. The etiology of AB is unknown, but the molecular pathogenesis has recognized mutations and altered gene expression levels in some ABs [1, 2, 3, 4]. Viruses can modulate the cellular DNA or its genetic activity both via direct and indirect actions, for example, via oncogenes, epigenetics, microRNA, or by chronic inflammation [5]. The possible role of a viral background of ABs has not been studied thoroughly regardless of the enormous microbial load and importance within other tumors of the anatomical area. The existing few studies have focused mainly on human papilloma virus and Epstein–Barr virus (EBV) [6, 7, 8, 9, 10], with conflicting results, potentially due to contamination issues from the surrounding tissues.

In the recent decade, new viruses have been recognized by means of modern molecular techniques, exemplified by new members within polyomaviruses and parvoviruses. It has been recognized that although some viruses can be identified as oncogenic, others may affect the tumor formation indirectly, for example, by influencing the immune system and microenvironment. This study was designed to explore broad‐scale virus presence in ABs, focusing on 30 herpes‐, parvo‐, and polyomaviruses (Table 1).

**TABLE 1:** Viruses analyzed in the study by qPCR and Luminex multiplex.

Herpesviruses (qPCR) [11]	Parvoviruses (qPCR) [12, 13]	Polyomaviruses (Luminex multiplex) [14]
Herpes simplex 1	Human parvovirus B19	BK polyomavirus
Herpes simplex 2	Human bocavirus 1	JC polyomavirus
Varicella Zoster	Human bocavirus 2	KI polyomavirus
Epstein–Barr virus	Human bocavirus 3	WU polyomavirus
Cytomegalovirus	Human bocavirus 4	MC polyomavirus
Human herpesvirus 6A	Cutavirus	Human polyomavirus 6
Human herpesvirus 6B	Tusavirus	Human polyomavirus 7
Human herpesvirus 7	Bufavirus	TS polyomavirus
Kaposi sarcoma virus		Human polyomavirus 9
		MW polyomavirus
		STL polyomavirus
		Human polyomavirus 12
		NJ polyomavirus

HUS Ethics Committee (151/13/03/02/2015) approved the study and Helsinki University Hospital Internal Review Board (10041/06.01.03.01/2012) granted the research permit. The principles of the Declaration of Helsinki and its later amendments have been followed within this study. The patients were treated at the Head and Neck Center, Helsinki University Hospital, Helsinki, Finland. Clinical information, including age, sex, and location, was obtained (Table 2). Eleven patients, treated during 1996–2015, with adequate, non‐decalcified formalin‐fixed, paraffin‐embedded (FFPE) tumor samples available (*n* = 11), were included in the analysis. The paraffin was dissolved, total DNA extracted, and viral DNA amplified with in‐house multiplex quantitative (q)PCR assays for 9 human herpesviruses and 8 human parvoviruses; and a multiplex PCR coupled with a Luminex‐based detection system for 13 polyomavirus analysis were performed, as earlier described [14, 15].

**TABLE 2:** Clinical, histological and cell quantity for each patient with ameloblastoma.

ID	Sex	Age	Histological classification	Anatomic location	Primary (P)/recurrence (R)	Cell quantity (RNASE‐P copies/mL)	Cell quantity (cells/mL)
**AB1**	F	71	Follicular/granular	Maxilla	R	2.4 10^3^	1.2 × 10^3^
**AB2**	M	86	Plexiform	Maxilla	R	1.8 10^4^	8.9 × 10^3^
**AB3**	M	79	Follicular, achantomatous	Maxilla	P	0.1 10^1^	2.07 × 10^1^
**AB4**	M	63	Follicular	Maxilla	P	0	No cells
**AB5**	M	83	Follicular	Maxilla	R	2.0 10^3^	9.9 × 10^2^
**AB6**	M	59	Plexiform, follicular	Maxilla	P	3.5 10^4^	1.8 × 10^4^
**AB7**	F	67	Plexiform, follicular	Mandible	R	2.5 10^4^	1.3 × 10^4^
**AB8**	M	73	Follicular	Mandible	P	2.6 10^4^	1.3 × 10^4^
**AB9**	M	87	Follicular, plexiform	Mandible	P	3.7 10^2^	1.9 × 10^2^
**AB10**	F	66	Not available	Mandible	R	4.6 10^3^	2.3 × 10^3^
**AB11**	F	31	Basaloid, achantomatous	Mandible	P	1.5 10^3^	7.5 × 10^2^

Molecular biology grade water tested negative as controls. Ten‐fold diluted plasmids (10^1^–10^6^) of each virus served as PCR standards and positive controls. The human reference gene *RNa*se *P* served as control for DNA quality and human cell quantity. One tumor was repeatedly *RNase P* negative suggesting unsuccessful DNA extraction. The mean *RNase P* qPCR result was 5.7 × 10^3^ copies/µL among the samples. All 30 viruses tested negative in all AB samples in duplicate analysis.

ABs originate from odontogenic epithelium entrapped within jaws or in the adjacent soft tissues. Therefore, it can be hypothesized that due to the intact location, the contact to microbial pathogens is minimal and hence a viral etiology is unlikely. In the recent few decades, two studies with rather small cohorts reported EBV positivity in approximately 25%–50% of ABs and a 63% CMV positivity in one study [8, 16, 17]. However, our results are supported by a larger cohort comprising 80 ABs, where no EBV positivity was detected by IHC [6]. In healthy oral mucosa, the prevalence of EBV is approximately 25%, and of CMV 10% [18].

Previously we have demonstrated the efficient use of FFPE tumor samples in virus DNA screening [15, 19, 20, 21]. The other studies utilized FFPE samples as well, so the conflicting results are not explained by different tumor treatment. However, our method of sampling differs from those of used in other PCR studies. We harvested tumor samples by taking them by punch biopsy. This aids to exclude any other possible tissue material in the tissue block surrounding the AB. In studies, where samples are harvested in the classical way by taking sections of the FFPE blocks (such as in the study by Alsaegh *et al*.), surrounding tissue, including, for example, epithelial tissue, may possibly cause false positive PCR results. Both multiplex PCR assays, the real‐time qPCR and the qualitative PCR‐coupled Luminex‐bead methods [14] offer extremely sensitive and specific virus detection. Indeed, even if all our viruses were negative in this material, we have obtained many PCR‐positive results with other tissue samples [15, 20, 21], and all our virus plasmids were positive down to 10 copies/µL (Table 2).

We investigated the presence of 30 DNA viruses within AB, being able to expand the limited knowledge on virus involvement in disease. Our results are the first to report on most human herpesviruses, parvoviruses, and polyomaviruses investigated in this tumor entity. No such virus DNA was present in the tumors of our cohort. We conclude that a relevant role of the investigated viruses within AB is less likely.

## AUTHOR CONTRIBUTIONS

**Conception and design**: Jaana Hagström, Jetta Kelppe, Maria K. Jauhiainen, Saku T. Sinkkonen, Maria Söderlund‐Venermo, Antti A. Mäkitie. **Acquisition or analysis of data**: Maria K. Jauhiainen, Jetta Kelppe, Jaana Hagström. **Interpretation of data**: Maria K. Jauhiainen, Maria Söderlund‐Venermo. **Drafting the article**: Maria K. Jauhiainen, Jetta Kelppe. **Revision of the article**: Maria K. Jauhiainen, Jetta Kelppe, Maria Söderlund‐Venermo, Antti A. Mäkitie, Saku T. Sinkkonen, Jaana Hagström.

## CONFLICT OF INTEREST STATEMENT

The authors declare no conflicts of interest.
